# Prioritizing population-based nutrition-related interventions to prevent and control hypertension in Iran: a multi-criteria decision-making approach

**DOI:** 10.1186/s12874-022-01761-z

**Published:** 2022-11-16

**Authors:** Soghra Aliasgharzadeh, Mehrangiz Ebrahimi-Mameghani, Reza Mahdavi, Hossein Karimzadeh, Leila Nikniaz, Jafar Sadegh Tabrizi, Fathollah Pourali

**Affiliations:** 1 Student Research Committee, School of Nutrition and Food Sciences, Tabriz University of Medical Sciences, Tabriz, Iran; 2 Social Determinant of Health Research Center, School of Nutrition and Food Sciences, Tabriz University of Medical Sciences, Tabriz, Iran; 3 Nutrition Research Center, Tabriz University of Medical Sciences, Tabriz, Iran; 4 Department of rural planning, Faculty of Planning and Environmental Sciences, University of Tabriz, Tabriz, Iran; 5 Tabriz Health Services Management Research Center, Tabriz University of Medical Sciences, Tabriz, Iran; 6 Community Nutrition Department of Public Health Deputy, Tabriz University of Medical Sciences, Tabriz, Iran

**Keywords:** Multi-criteria decision-making, Analytic hierarchy process, Delphi technique, Nutrition-related intervention, Hypertension, Non-communicable diseases

## Abstract

**Background:**

Numerous nutrition-related policy options and strategies have been proposed to tackle hypertension and other risk factors of non-communicable diseases (NCDs). In this study, we developed a comparative analysis using a multi-criteria decision-making (MCDM) model for prioritizing population-based nutrition-related interventions to prevent and control hypertension in Iran.

**Methods:**

We employed a combination of Delphi technique and Analytic Hierarchy Process (AHP) method as the methodological tool to prioritize decision alternatives using multiple criteria. The prominent assessment criteria and intervention strategies were derived using a literature review, focus group discussion (*n* = 11), and a 2-round modified Delphi technique with specialists and experts involved in different stages of health policy-making (round 1: *n* = 50, round 2: *n* = 46). Then, the AHP was used to determine the weightage of the selected interventions and develop the decision-making model. The sensitivity analysis was performed to test the stability of the priority ranking.

**Results:**

Nine alternative interventions were included in the final ranking based on eight assessment criteria. According to the results, the most priority interventions to prevent and control hypertension included reformulation of food products to contain less salt and changing the target levels of salt in foods and meals, providing low-sodium salt substitutes, and reducing salt intake through the implementation of front-of-package labeling (FOPL). The results of the sensitivity analysis and a comparison analysis suggested that the assessment model performed in this study had an appropriate level of robustness in selecting the best option among the proposed alternatives.

**Conclusion:**

MCDM techniques offer a potentially valuable approach to rationally structuring the problem, along with the opportunity to make explicit the judgments used as part of the decision-making model. The findings of this study provide a preliminary evidence base to guide future decisions and reforms aiming to improve appropriate population-based interventions for tackling hypertension and other risk factors of NCDs.

## Background

Hypertension is one of the most preventable causes of premature morbidity and mortality worldwide. It is a major risk factor for stroke, chronic kidney disease, congestive heart failure, myocardial infarction, and peripheral artery disease [1–3]. Researchers have estimated that hypertension accounts for approximately 9.4 million premature deaths and about 7% of global disability-adjusted life years (DALYs) worldwide annually [4]. In the past two decades, the prevalence of hypertension decreased moderately in high-income countries whereas it increased significantly in low and middle-income countries (LMICs) [5]. In Iran, according to findings of the population-based national STEPs surveys, the prevalence of hypertension increased from 14.66 to 32.03% between 2009 and 2021. The increasing prevalence of hypertension is attributed to population aging, and behavioral risk factors, such as unhealthy diet, lack of physical activity, harmful use of alcohol, excess weight, and exposure to persistent stress [6, 7]. Despite the high prevalence of hypertension, the rates of management, and control of high blood pressure are unsatisfactory and demand comprehensive strategies to improve this condition [8].

Clinical and population-based studies show that several components of the diet, such as fruits and vegetables, and foods high in saturated fats, trans fats, salt, and sugar, affect blood pressure [9, 10]. So, modifying these nutritional factors through population-based approaches can reduce the burden of hypertension [11]. Different strategies and policy options involving both upstream (or structural) and downstream (or agentic) interventions have been proposed to achieve this goal [11–13].

High-income countries have started to enact strong public health policies pertaining to healthy eating, such as mass media campaigns or limiting salt in processed foods to reduce the prevalence of hypertension in their populations [13]. For example, Finland initiated a comprehensive approach to reduce salt intake in the late 1970s using mass media campaigns, labeling legislation, and voluntary reformulation by the food industry. A one-third decrease in salt intake was accompanied by a decline in systolic blood pressure (SBP) and diastolic blood pressure (DBP) of 10 mmHg or more [14, 15]. In the United Kingdom, a combination of consumer awareness campaigns, agreed target settings, voluntary industry reformulation, labeling, setting lower salt targets for various food categories, and population monitoring of salt consumption resulted in the reduction of population salt intake by 1.4 g per day from 2003/2004 to 2011 [16]. In Denmark, tax on saturated fat in food products reduced fat intake by 10–15% [17]. Although the national capacity for implementing these interventions and programs have been inefficient in most developing countries, several upper and lower-middle-income countries have adopted some interventions for this regard. In Iran, several policies to tackle non-communicable diseases (NCDs) have been implemented in line with the World Health Organization (WHO) Global NCD action plan, including regulations on food labeling on salt and fat content and reducing the amount of salt in bread. However, the feasibility and effectiveness of these programs are not usually examined before and after the implementation, and the rates of management, and control of high blood pressure remains unsatisfactory [8, 18].

Given the limited public resources available to meet government priorities and objectives, identifying the most effective and feasible interventions and an efficient combination of them seems necessary [19]. In this respect, multi-criteria decision-making (MCDM) methods are used to make decisions about solving complex problems, such as evaluating and allocating resources, and selecting the most appropriate policies from a set of predetermined alternatives [20–22]. The application of these approaches has become increasingly popular in medical and public health decision-making settings, both at the policy and patient levels [23–25]. The essence of MCDM is to rank all the alternatives and select the most desirable one by applying specific approach taking into consideration different evaluation criteria [26]. In this field, weighing a particular criterion plays a significant role as they provide the importance of different criterion [26]. Various MCDM techniques have been developed to estimate the weights of the criteria close to the preferences of the decision maker. Each MCDM method has been developed with different advantages and disadvantages, though the researchers usually select an approach based on the nature and intricacy of the problem [27, 28]. There are no criteria for the effectiveness of weighing methods [27, 29]. From the literature review, it can be clearly noticed that different mathematical techniques have mainly been employed for two purposes: (a) determining the relative weights of the considered criteria by evaluating one against the others, and (b) ranking the candidate alternatives based on the accumulative score with respect to each criterion [30]. Recently, Analytical Hierarchy Process (AHP), Best-Worst Method (BWM), Level Based Weight Assessment (LBWA), and Full Consistency Method (FUCOM) have been increasingly applied in different works because of the ability to determine the degree of consistency. A more recently defined approach is FUCOM developed by Pamučar et al. to estimate the weights of the criteria using a crisp scale [31]. In this method, the criteria are initially ranked, then compared with other criteria with respect to the first rank. The main advantage of FUCOM is that it eliminates the problem of redundancy of pairwise comparison, which is present in some subjective models for determining the weight of the criteria [31, 32]. However, one disadvantage of this method is that the process of calculation is complicated compared to other methods such as BWM [32]. Since the FUCOM is a quit new method, there are lack of more studies to verify the validation of this model through literature review [32]. LBWA model also allows for the calculation of weight coefficients with a minimum number of criteria in pairwise comparison [33]. Another advantage of LBWA method is the simple and rational mathematical algorithm, which does not become complex with increasing the number of criteria in the multicriteria model [34]. But this method cannot handle uncertainty, and is not applicable in a group decision-making environment [35]. AHP and BWM are based on a systematic pairwise comparison of the decision criteria [36]. Contrary to the AHP, in the BWM, the most important (the best) and the least important (the worst) criteria are identified first by the decision-maker [36]. Then, a comparison is made between each of these two criteria (best and worst) with the other ones. The main advantages of BWM method include achieving better consistency in pairwise comparisons and more reliable weight results due to the use of fewer comparison data [27, 37]. However, this model is unacceptable to many researchers as solving non-linear models makes the application of the BWM significantly more complex [33]. Also, in numerous real-world problems, there are situations in which defining one unique best and/or worst criterion/criteria is difficult [38].

The AHP technique, developed by Saaty in late 1970, is the most widely used and effective MCDM approach in complex environments [39, 40]. The method uses pairwise comparisons of predefined set of criteria/alternatives for alternatives’ prioritization. The most prominent advantage of the AHP include structuring complex decision problems into a hierarchy of interrelated elements (including goals, criteria, and possible alternatives), comparing the importance of various criteria, and finally assessing decision alternatives under the existing structure [41, 42]. This method enables decision-makers to collaboratively translate independent subjective judgments into ratio scale in a rational manner and increase the transparency of the decision-making process. The significant challenge to the pairwise comparison method is that a large number of comparisons results in an increase in inconsistency, especially in cases of a large number of criteria. According to some studies, it is almost impossible to perform completely consistent pairwise comparisons in the AHP method if there are more than nine criteria; this is often overcome by dividing the criteria into sub-criteria [43, 44]. AHP has already been applied in various fields, including marketing, research and development project selection and resource allocation, logistics, transportation, and other decision-making contexts [45–50]. For instance, Alossta et al. solved the optimal location selection problem through the combined AHP method [51]. In the field of logistics, Karamaşa et al. identified priority factors for the impact of logistics outsourcing based on Neutrosophic AHP [49]. The technique was also used in health care and medical decision-making. The usefulness of AHP to support group decisions in the medical and health care domains has been established in several previous studies [48, 52, 53]. The results of a systematic review indicated that AHP is a promising support method for shared decision-making between patients and physicians, evaluation and selection of diagnoses and treatments, development of clinical guidelines, and evaluation and prioritizing of health care policies and technologies [54]. Recently Byun et al. applied the AHP model to prioritize community-based interventions for sustainable management of hypertension and diabetes [55].

Although several efforts in the area of nutrition have been made for the prevention and management of hypertension in developing countries such as Iran, population-based studies have shown that control and preventive measures for high blood pressure levels are far from the optimal level [56]. Evidence indicated that the formulation of these policies faces barriers and challenges that restrict achieving the desired effects on the intended outcomes [57–59]. There are several possible reasons for the failure and ineffectiveness of these programs, including a focus on single criteria for priority setting (such as effectiveness, cost-effectiveness, etc.) and ignoring the other considerations such as feasibility, acceptance, sustainability, and the relative importance of each criterion [60, 61]. For example, in 2014, the Ministry of Health, Treatment and Medical Education (MoHME) formulated the national guidelines for Healthy School Canteen (HSC), based on the available evidence regarding the high consumption of unhealthy and nutrient-poor foods by children and availability of such foods in schools [62]. Based on the HSC guideline, all schools in Iran should provide healthy foods and drink choices in their canteens and limit selling unhealthy items [57, 62]. Two recent studies evaluated the implementation of this bylaw and provided evidence on the failure to achieve the desired result [63, 64]. There are several main reasons behind the poor compliance of schools with this guideline, including inadequate physical and economic infrastructure of schools to set up standard canteens, the high price and limited availability of healthy alternatives, and conflict of interest between the actors [64, 65]. So, given the limited public resources available to meet government objectives, choosing an appropriate approach or a combination of approaches should be made by considering all the attributes of the interventions and multiple criteria for assessing them (e.g., effectiveness, implementation costs, acceptance, feasibility, sustainability, etc.) [61, 66]. Will the policy advance equity? Is there sufficient evidence for impact? Is the policy feasible, practical, politically acceptable, and legal to implement? Will the implementation of this policy have a temporary or permanent effect? The answers to these questions will differ across policy options and contexts with different socioeconomic status, technology advancement, cultural futures, and consumption patterns. Therefore, a one-size-fits-all approach will not work; determining the best policy options requires simultaneous consideration of the specific social and political context of a population segment.

To our knowledge, there is a lack of research that pays attention to prioritizing nutrition-related intervention by considering key policy criteria using MCDM approaches in Iran. Hence, this study aimed to address the gap in decision-making by implementing a comparative analysis for prioritizing population-based nutritional interventions to prevent and control the prevalence of hypertension in Iran using AHP method.

Although there are new and useful decision-making methods for MCDM, AHP still has many advantages as follows: (i) AHP is concise and easy to understand and grasp for decision-makers and scholars; (ii) Since it has been applied in various domains, especially in health care recently, the validity of this model can be verified through literature review; (iii) It uses hierarchical structures to model problems and has less equalizing bias; and (iv) This method has strong practicality and can be used in combination with other methods to compensate for its shortcomings [67–71]. Strategy prioritization through a transparent and systematic approach that key stakeholders take into account the available strategy options and all relevant criteria simultaneously, and relative weighting scheme in accordance with the current socioeconomic status of society can help to develop more appropriate measures to achieve the goals of national action plans for prevention and control of NCDs.

## Methods

**Fig. 1:**
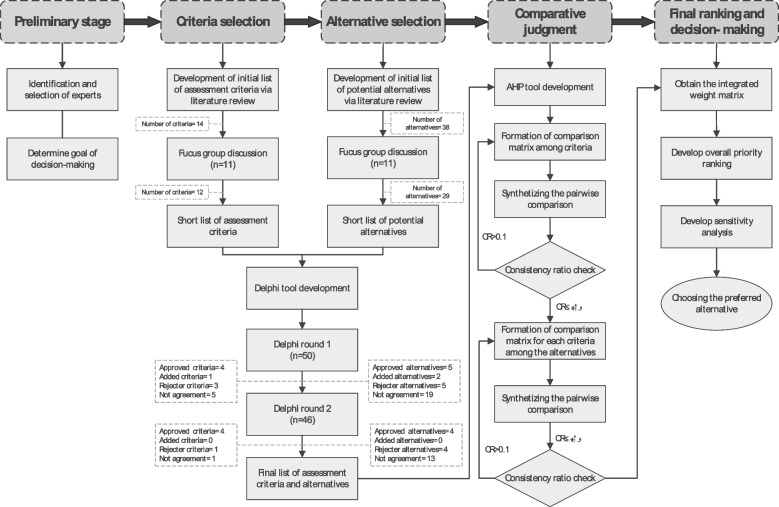
Flowchart of the study process for prioritizing population-based nutrition interventions for the prevention and control of hypertension

We employed a combined Delphi-AHP method as an analytical tool to prioritize decision alternatives using multiple criteria. While the Delphi technique was used at the preliminary stages of research to develop a shortlist and identify the more prominent assessment criteria and intervention strategies, the AHP was used to determine the weightage of the selected interventions and develop the decision-making model. A diagram that outlines the current study scheme for intervention prioritization is depicted in Fig. 1 and describe below.

### Recruitment of expert panel

in this study, we defined experts as individuals involved in various stages of health policy process (agenda setting, formulation, adoption, implementation, and evaluation) at global, national, and regional levels, as well as researchers and specialists with substantial knowledge and information on such fields as nutrition and food policy and NCDs control and prevention. The inclusion criteria for the participants were as follows: at least 5 years of work experience in the related field; no direct conflict of interest with the study topic; willingness to participate in the study; and sufficient time to answer the questions.

There are no general or specific rules for an optimal panel size in Delphi and AHP studies [72, 73]. However, to determine the panel size, it has been proposed to consider the aim of the investigation, the homogeneity or heterogeneity of the sample, the complexity of the problem, and the resources available [74]. Accordingly, 50 participants were recruited in the Delphi study through purposive and snowball sampling methods. In this way, after reviewing related documents, literature and websites and following the guidance of five key stakeholders in the field of health policy-making in Iran, a list of panel experts was prepared. Two of these stakeholders were policymakers at the national level from MoHME, two were the members of the Non-Communicable Diseases Department of the Provincial Health Center, who were well acquainted with the people and organizations involved in this policy field, and one stakeholder was a university professor at the provincial level with high experience and familiarity with organizations that cooperate in this policy area. Considering the selected prominent criteria and intervention strategies in the Delphi process, the number of samples was adjusted and 29 experts were selected for the AHP survey. Initially, an electronic invitation letter explaining the goal and protocol of the study was sent to all the identified experts. After obtaining consent from participants, the questionnaires developed in each stage of study were sent to them via email, subsequently. Table 1 shows the general characteristics, and expertise profiles of each step.

**Table 1:** General characteristics of participants in each stage of the study

	Delphi round 1 (*n* = 50)	Delphi round 2 (*n* = 46)	AHP (*n* = 29)
**Gender**			
male	27	26	17
female	23	20	12
**Age** (years)			
30–39	13	12	8
40–49	17	17	11
50–59	18	15	9
≥60	2	2	1
**Highest education**			
Bachelor	5	5	2
Master	10	10	6
Doctor of Medicine	9	7	4
Doctor of Philosophy	26	24	17
**Professional experience** (years)			
5–10	11	10	5
11–15	9	8	7
16–20	11	11	6
21–25	13	12	8
≥26	6	5	3
**Job position**			
Professor of the University of Medical Sciences	10	10	8
Office of Community Nutrition Improvement	8	7	4
Food and Drug Organization	4	4	3
Professor of the Faculty of Agriculture	1	1	0
Managing Director of Food Production Factory	2	2	1
Islamic Republic of Iran Broadcasting	1	1	0
Department of Population and Family Health of Provincial Health Center	3	2	1
Department of Non-Communicable Diseases of the Provincial Health Center	6	5	4
School Health Department of the Provincial Health Center	2	2	1
Department of Health Education of the Provincial Health Center	1	1	0
Cardiologist	2	2	1
Provincial Office of Education	2	2	0
The Research Department of Food and Nutrition Policy and Planning, National Nutrition and Food Technology Research Institute	3	3	2
The Research Department of Food Science and Technology, National Nutrition and Food Technology Research Institute	4	3	2
Health Services Management Research Center	1	1	2
**Specialty**			
Food science and technology	5	5	4
Nutrition sciences	10	9	6
Nutrition and food policy	4	4	2
Nutritional epidemiology	0	0	1
Health policy	5	4	2
Health care service management	2	2	2
Health economic	2	2	2
Public health	3	3	1
Health education and promotion	3	3	1
Public health in nutrition	5	5	3
Family health	2	2	1
Cardiology	2	2	1
Medicine	5	4	2
Pharmacology	2	1	1

### Implementation of AHP

**Fig. 2:**
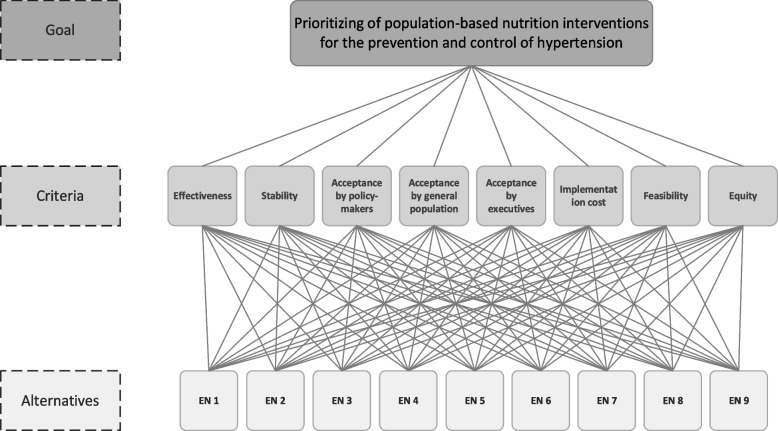
Hierarchical structure of the AHP model for prioritizing population-based nutrition interventions for the prevention and control of hypertension

The steps for applying the AHP approach are shown in Fig. 2 and detailed below:

#### First step: defining the decision goal

We determined the goal of the decision as the need to prioritize population-based nutrition interventions for prevention and control of hypertension so that it can be used in decision-making at national levels. This step was discussed in more detail in Introduction section.

#### Second step: determining the criteria for intervention assessment

The set of criteria consists of all considerations that should be satisfied to prioritize and select the options. As a general rule, the criteria must be exhaustive in order not to forget any aspect playing an important role in the prioritization. Moreover, they must be parsimonious so that the essential aspects are considered. For this purpose, we performed a comprehensive electronic search to develop initial judgment criteria for making intervention assessments. The identified criteria were then discussed and scrutinized by the panel of experts (four from the research team and seven other specialists) in a focus group session to reach a consensus on the list of assessment criteria. In the next step, two rounds of modified Delphi process were conducted to finalize the criteria. A Delphi process is defined as a multistage survey that ultimately attempts to achieve consensus on an important issue, and its basic characteristics include anonymity, iteration of rounds, controlled feedback, and statistical aggregation to create a group response [75, 76]. In this method, unimportant criteria can be identified and eliminated from further consideration, and at the same time, any important missed criteria can be added. In the first round, after fully explaining the content and the purpose of the research through electronic invitation letter and obtaining the consent, the Delphi survey questionnaire was sent to the panel experts via e-mail. Each expert was asked through a questionnaire to specify the importance of each evaluation criteria using a 10-point Likert scale ranging from 1 (not important) to 10 (very important). All the potential criteria were listed along with a short description of them. Also, the respondents were provided with an open text box to add any possible important items missing in the list. A consensus was achieved if the criteria, including a mean of at least 7.0 and a SD of ≤1, were met. Regarding the statements for which a consensus was not achieved, feedback and comments were provided to adjust the statement for the following round. Furthermore, any additional items proposed in the free-text comment box in round 1 were discussed by the research team and included for rating in round 2 if the majority of team members agreed. In the second round each panel member was provided with the summary results of the first round, including their previous judgment for each item and aggregation of all individual answers. This enabled each participant to consider their own responses with respect to the group opinion. The answers were analyzed in the same manner as round one.

The final criteria proposed by the expert group to be included in the AHP process consisted of eight items as follows: ‘effectiveness’, ‘implementation costs’, ‘acceptance by policy-makers’, ‘acceptance by population’, ‘acceptance by executives’, ‘feasibility’, ‘sustainability’, and ‘equity’. The description of the evaluation criteria are presented in the Table 2.

**Table 2:** Decision - making criteria

Criteria	Considerations
Effectiveness	To what extent will the intervention strategy produce the desired outcome in real - world settings. The extent to which an intervention achieves the desired outcome in real - world settings/context.
Implementation costs	What are the costs related to developing and executing the intervention strategy?
Acceptance by policy-makers	Is the intervention strategy politically acceptable? To what extent will the program be accepted by decision-makers?
Acceptance by population	To what extent will the intervention strategy be accepted by the target community?
Acceptance by executives	To what extent will the intervention strategy be accepted by the executives?
Feasibility	Is there adequate capacity to implement the intervention strategy? Is the strategy feasible in the current context?
Sustainability	Is ongoing capacity and infrastructure required for the intervention strategy to continue?
Equity	Does intervention strategy reach high priority groups? Is the strategy impact evenly distributed or does it have high impact on a few people or a low impact on many?

#### Third step: determining alternative strategies

A comprehensive literature review was performed to develop an initial list of population-based interventions to reduce the intake of salt, sugar, trans-fatty acids, and saturated fatty acids, and increase the consumption of fruits and vegetables. The literature search was conducted in the following electronic databases: Web of Science, Medline, EMBASE, PubMed, Scopus, Cochrane Library, Science Direct, Global Health Council, Health Action International, and Management Sciences for Health (MSH). The pertinent grey literature and qualitative documents, such as books, and policy reports, were obtained via Google’s online search engine, Open Grey, WHO and regional office databases and websites, as well as governmental websites. Accordingly, a list of 38 intervention strategies that could potentially address the problem was developed, and details were added by the research team for further consideration. The candidate intervention strategies were subsequently discussed separately by the panel of experts (four from research team and seven other specialists) in focus group discussion session to obtain recommendations for which interventions should be shortlisted for the subsequent Delphi survey. In the following step, two rounds of the modified Delphi process were performed as a criteria selection step, with the participation of the same experts to finalize the intervention strategies (see the Determining the criteria for intervention assessment section). The final set included nine intervention strategies as the decision alternatives.

#### Fourth step: performing pairwise comparisons to derive weights for hierarchical elements

Pairwise comparisons were made by comparing the elements of every hierarchy level relative to each element on the next higher level. This was carried out by asking the experts to judge each pair of decision criteria with respect to the goal using Saaty’s discrete scale [77], as presented in Table 3. The scale ranges from 1 to 9, where one implies that the two elements are equally important, and a score of 9 implies that one element is extremely more important than the other. In a similar manner, another matrix was again constructed to determine the relative performances of pairs of intervention strategies with respect to their contribution towards fulfilling each given criterion. For these pairwise comparisons, a score of 9 indicated an extreme preference for one alternative over another (Table 3). The pairwise comparison matrixes can be expressed as Eq. (1). The score of a_*ij*_ in the pairwise comparison matrix symbolizes the relative importance of the element on the row (*i*) over the element on column (*j*).

1 $$\textrm{A}=\left[{a}_{ij}\right]=\left[\begin{array}{cccc}1& {a}_{12}& \cdots & {a}_{1n}\\ {}^{1}\!\left/ \!{}_{{a}_{12}}\right. & 1& \cdots & {a}_{2n}\\ {}\vdots & \vdots & 1& \vdots \\ {}^{1}\!\left/ \!{}_{{a}_{1n}}\right. & {}^{1}\!\left/ \!{}_{{a}_{2n}}\right. & \cdots & 1\end{array}\right]$$

**Table 3:** Pair wise comparison scale for AHP preference

Intensity of judgments	Numerical rating
Extremely preferred, desired, or important	9
Very strongly preferred, desired or important	8
Strongly preferred, desired or important	7
Moderately preferred, desired or important	6
Sufficiently preferred, desired or important	5
Preferred, desired or important	4
Slightly preferred, desired or important	3
Hardly preferred, desired or important	2
Equally preferred, desired or important	1

The matrix has reciprocal properties (*a*_*ij*_ = 1/ *a*_*ij*_). In the AHP method, after forming all pairwise comparison matrices, the vector of weights (*w* = [*w*_1_, *w*_2_, …, *w*_*n*_]) is calculated based on Satty’s eigenvector method [78]. To this end, first the pairwise comparison matrix, A = [*a*_*ij*_]_*n* × *n*_, is normalized with Eq. (2) and then the weights are calculated using Eq. (3).

2 $${a}_{\textrm{i}j}^{\ast }=\frac{a_{ij}}{\sum_{i=1}^n{a}_{ij}}\kern0.5em \textrm{for}\ \textrm{all}\ j=1,2,\dots, n.$$

3 $${\displaystyle \begin{array}{cc}{w}_{\textrm{i}}=\frac{\sum_{j=1}^n{a}_{\textrm{i}j}^{\ast }}{n}\ & \textrm{for}\ \textrm{all}\ i=1,2,\dots, \textrm{n}.\end{array}}$$

As shown in Eq. (4), there is a relationship between the vector weights, *w*, and the pairwise comparison matrix A.

4 $$Aw={\lambda}_{max}w$$

Where *w* is the vector of the absolute values, and *λ*_*max*_ is the highest of the eigenvalues of the matrix A.

Due to the inconsistency of human judgments, while the values are allocated during the pairwise comparison process, AHP allows for the calculation of a measure of consistency, reflecting how logical each pairwise comparison is with regard to the remainder of comparisons performed by the same individual [41]. The CR is calculated by comparing the consistency index (CI) of the matrix in question with the consistency index of a random-like matrix (RI). RI is the random consistency index derived from numerous randomly generated reciprocal matrices. Therefore, the CR is determined by Eq. (5) and (6).

5 $$CI=\frac{\lambda_{max}-n}{n-1}$$

6 $$CR=\frac{CI}{RI}$$

From Saaty’s viewpoint, paired comparisons are consistent if the CR is less than or equal to 0.10. Otherwise, another cycle of reassessing of the paired comparisons is required until CR falls below 0.10 [41].

To obtain an aggregate measure of the pairwise comparisons of all individuals involved in the decision problem, the geometric mean of the individual assessments is calculated by Eq. (7).

7 $${a}_{\textrm{i}j}^{hp}=\sqrt[M]{\prod_{m=1}^M{a}_{\textrm{i}j}^m}$$

where ${a}_{\textrm{i}j}^m$ is an element of matrix A of an individual m (m = 1, 2, . . ., M), and ${a}_{\textrm{i}j}^{hp}$ is the geometric mean of all individuals ${a}_{\textrm{i}j}^m$.

#### Fifth step: calculating the composite priority weights for decision alternatives

In order to rate the decision alternatives, the weights generated in the previous step are synthesized into a composite weight for each intervention strategy. In other words, the overall priority of an intervention is determined by the weighted sum of the relative preferences for the intervention with respect to each decision criterion. To weigh these relative preferences, they are multiplied by the importance of each criterion.

### Validation of results

To confirm the accuracy of the results and to validate the robustness and applicability of the proposed approach, we performed sensitivity analysis of the threshold value and comparative analysis of the methods. Sensitivity analysis was conducted to determine the impact of altering the weights assigned to each criterion on the final ranking of the alternatives. As health care decisions may be inherently unstable, sensitivity analysis is a vital part of such decision processes [79]. Sensitivity analysis allows the decision results to be verified [80].

The AHP was conducted using the Expert Choice (EC) 11 software (Arlington, Virginia, USA) and SPSS software version 23.0. Also, the study received ethical approval by the Ethics Committee of Tabriz University of Medical Sciences (code: IR.TBZMED.REC.1398.1158).

## Results

Out of 55 contacted experts, 50 experts responded and completed the first round of the Delphi survey (response rate: 91%). The second round of the survey was completed with the participation of 46 experts (response rate 92%). We only invited respondents from round one to participate in the second round. Furthermore, out of the 33 experts surveyed, 29 (88%) individuals provided complete responses to the AHP part of the survey. Table 1 provides an overview of the participant demographics and professional background.

**Fig. 3:**
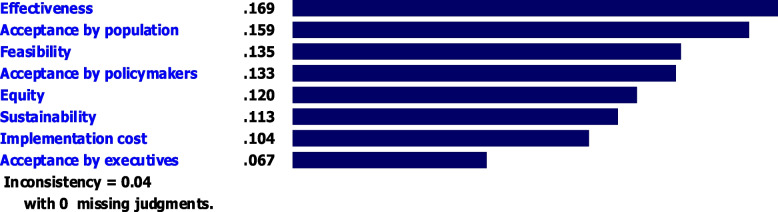
Overall priority weights and ranking of assessment criteria using AHP method

The results of the prioritization of the assessment criteria from the experts’ viewpoint is presented in Fig. 3. Accordingly, effectiveness was the most important criterion to select an intervention strategy to tackling hypertension with a mean priority score of 0.169, followed by acceptance by general population (0.159), feasibility (0.135), acceptance by policy-makers (0.133), equity (0.120), sustainability (0.113), implementation cost (0.104), and acceptance by executives (0.067).

**Fig. 4:**
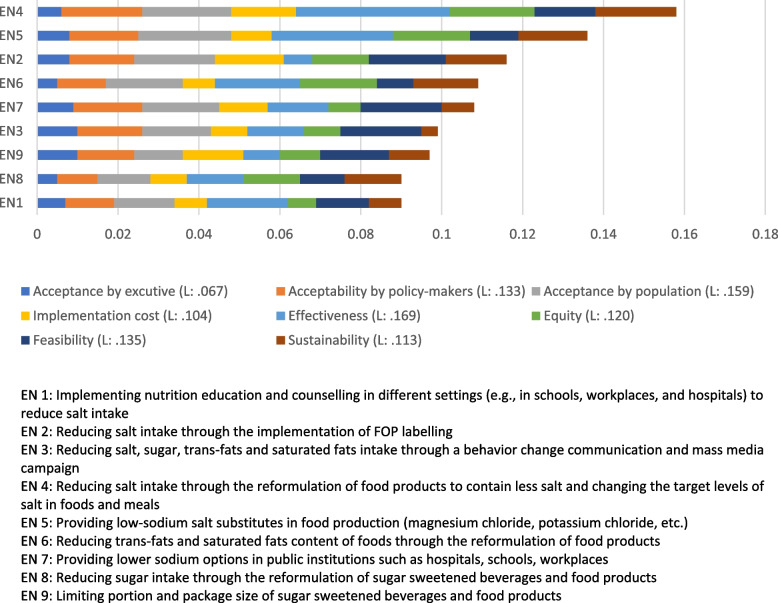
Prioritization of intervention strategies for the prevention and control of hypertension based on eight criteria using AHP method

Figure 4 depicts the prioritization of intervention strategies to prevent and control hypertension based on the eight mentioned criteria. Accordingly, ‘*reformulation of food products to contain less salt and changing the target levels of salt in foods and meals*’ (0.158) was identified as the intervention with the highest priority. It was then followed by ‘*providing low-sodium salt substitutes in food production (magnesium chloride, potassium chloride, etc.)*’ (0.136), ‘*reducing salt intake through the implementation of FOP labelling*’ (0.116), ‘*reducing trans-fats and saturated fats content of foods through the reformulation of food products*’ (0.109), and ‘*providing lower sodium options in public institutions such as hospitals, schools, workplaces*” (0.108). Meanwhile, the following were identified as the interventions with the lowest priority: ‘*reducing salt, sugar, trans-fats and saturated fats intake through a behavior change communication and mass media campaign’* (0.099), ‘*limiting portion and package size of sugar sweetened beverages and food products’* (0.097), ‘*reducing sugar intake through the reformulation of sugar sweetened beverages and food products*’ (0.0.091), and ‘*implementing nutrition education and counselling in different settings (e.g., in schools, workplaces, and hospitals) to reduce salt intake*’ (0.090).

**Fig. 5:**
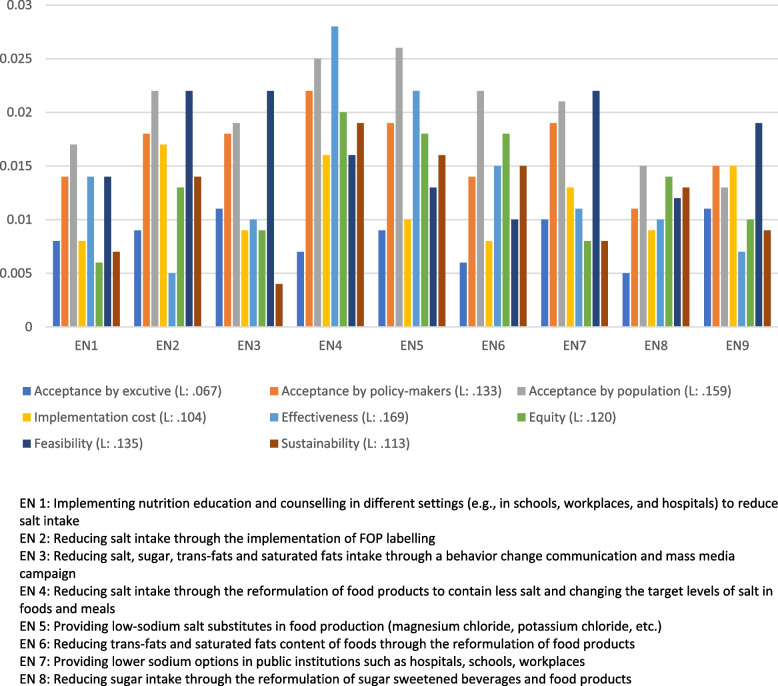
Priorities and rankings of intervention strategies for the prevention and control of hypertension with respect to each criterion using AHP method

Table 4 summarizes the results of direct comparisons of intervention strategies based on each criterion. As can be seen, the expert panel rated ‘*reducing salt intake through the reformulation of food products to contain less salt and changing the target levels of salt in foods and meals*’ as the intervention with the highest priority in terms of effectiveness, equity, sustainability, and acceptance by policy-makers, while ‘*providing low-sodium salt substitutes in food production (magnesium chloride, potassium chloride, etc.)*’ was identified as the intervention with the highest priority in terms of acceptance by population. Regarding the implementation costs, ‘*reducing salt intake through the implementation of FOP labelling*’ was rated as the most favorable intervention. Furthermore, from the experts’ point of view, ‘*providing lower sodium options in public institutions such as hospitals, schools, and workplaces*’ and ‘*limiting portion and package size of sugar-sweetened beverages and food products’* were identified as the most feasible and executives accepted alternatives. Figure 5 depict the results of comparison of alternatives with respect to each criterion. The results of the consistency test of the comparison matrix from each of the panel experts were all smaller than 0.1, and the overall CR value was 0.068, indicating good consistency in judgments.

**Table 4:** Priorities and rankings of intervention strategies for the prevention and control of hypertension with respect to each the main criteria

Alternative	Acceptance by executives (L: 0.067)	Acceptance by policymakers (L: 0.133)	Acceptance by population (L: 0.159)	Implementation cost (L: 0.104)	Effectiveness (L: 0.169)	Equity (L: 0.120)	Feasibility (L: 0.135)	Sustainability (L: 0.113)	Overall priority weight	Total rank
EN 1	0.007 (6)	0.012 (8)	0.015 (7)	0.008 (9)	0.020 (4)	0.007 (9)	0.013 (6)	0.008 (8)	0.090	9
EN 2	0.008 (5)	0.016 (4)	0.020 (3)	0.017 (1)	0.007 (9)	0.014 (5)	0.019 (3)	0.015 (4)	0.116	3
EN 3	0.010 (2)	0.016 (5)	0.017 (6)	0.009 (6)	0.014 (7)	0.009 (7)	0.020 (1)	0.004 (9)	0.099	6
EN 4	0.006 (7)	0.020 (1)	0.022 (2)	0.016 (2)	0.038 (1)	0.021 (1)	0.015 (5)	0.020 (1)	0.158	1
EN 5	0.008 (4)	0.017 (3)	0.023 (1)	0.010 (5)	0.030 (2)	0.019 (3)	0.012 (7)	0.017 (2)	0.136	2
EN 6	0.005 (8)	0.012 (7)	0.019 (4)	0.008 (8)	0.021 (3)	0.019 (2)	0.009 (9)	0.016 (3)	0.109	4
EN 7	0.009 (3)	0.017 (2)	0.019 (5)	0.012 (4)	0.015 (5)	0.008 (8)	0.020 (2)	0.008 (7)	0.108	5
EN 8	0.005 (9)	0.010 (9)	0.013 (8)	0.009 (7)	0.014 (6)	0.014 (4)	0.011 (8)	0.014 (5)	0.091	8
EN 9	0.010 (1)	0.014 (6)	0.012 (9)	0.015 (3)	0.009 (8)	0.010 (6)	0.017 (4)	0.010 (6)	0.097	7

Data is presented as weight (rank)

EN 1: Implementing nutrition education and counselling in different settings (e.g., in schools, workplaces, and hospitals) to reduce salt intake

EN 2: Reducing salt intake through the implementation of FOP labelling

EN 3: Reducing salt, sugar, trans-fats and saturated fats intake through a behavior change communication and mass media campaign

EN 4: Reformulation of food products to contain less salt and changing the target levels of salt in foods and meals

EN 5: Providing low-sodium salt substitutes in food production (magnesium chloride, potassium chloride, etc.)

EN 6: Reducing trans-fats and saturated fats content of foods through the reformulation of food products

EN 7: Providing lower sodium options in public institutions such as hospitals, schools, workplaces

EN 8: Reducing sugar intake through the reformulation of sugar sweetened beverages and food products

EN 9: Limiting portion and package size of sugar sweetened beverages and food products

### Sensitivity analysis

**Fig. 6:**
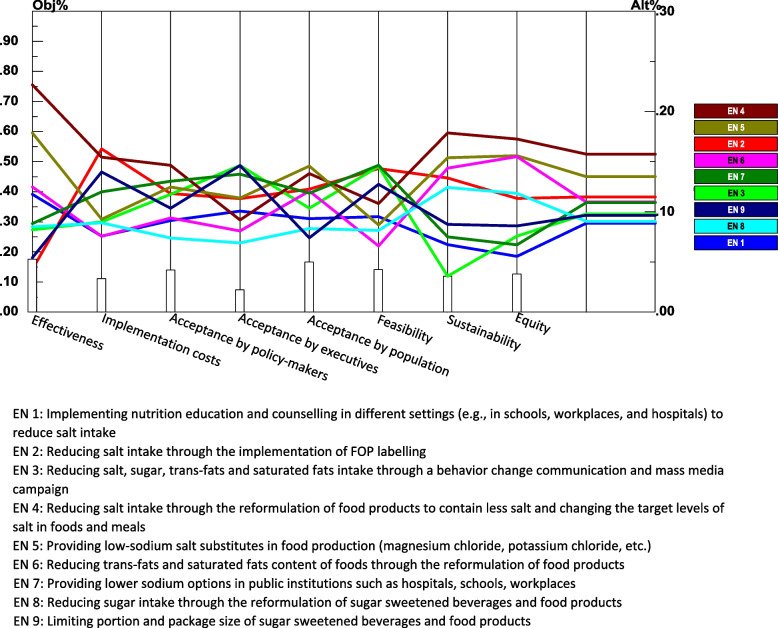
Performance sensitivity analysis for priority of intervention strategies for the prevention and control of hypertension

A sensitivity analysis was carried out to examine the robustness and stability of rankings expressed by experts. The performance sensitivity analysis graph (Fig. 6) shows how each alternative was prioritized relative to another alternative with respect to each criterion as well as overall. This investigation can help decision-makers to identify the strength and weakness of the alternatives. The following scenarios were performed by changing the weight of the criteria to verify the response in the chosen alternatives.

#### Scenario 1: the sensitivity analysis assuming equal weight to all assessment criteria

**Fig. 7:**
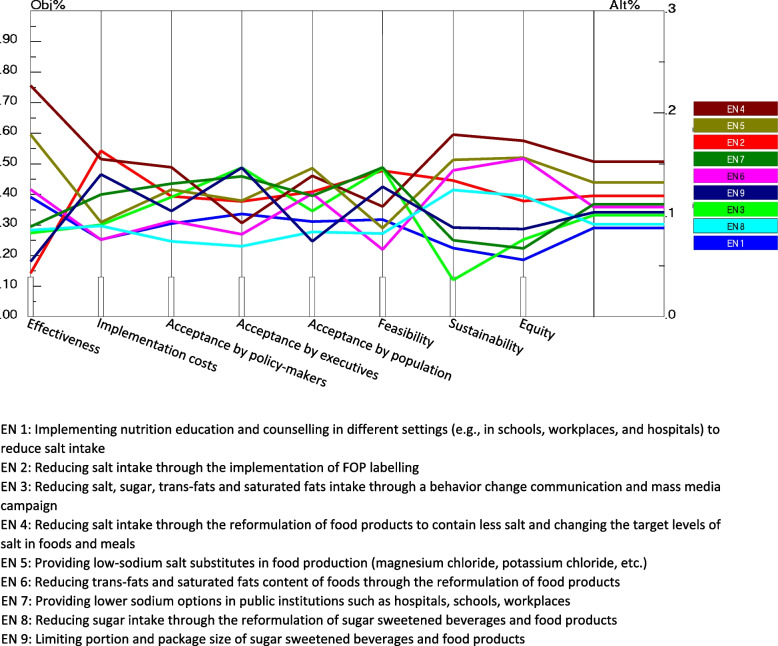
Performance sensitivity analysis for priority of intervention strategies when assuming equal weight to all assessment criteria

The analyses showed that the fourth and fifth ranks, as well as the sixth and seventh ranks were exchanged (Fig. 7). There was no change in the position of the other alternatives.

#### Scenario 2: the sensitivity analysis by slightly altering the weights assigned to ‘sustainability’

**Fig. 8:**
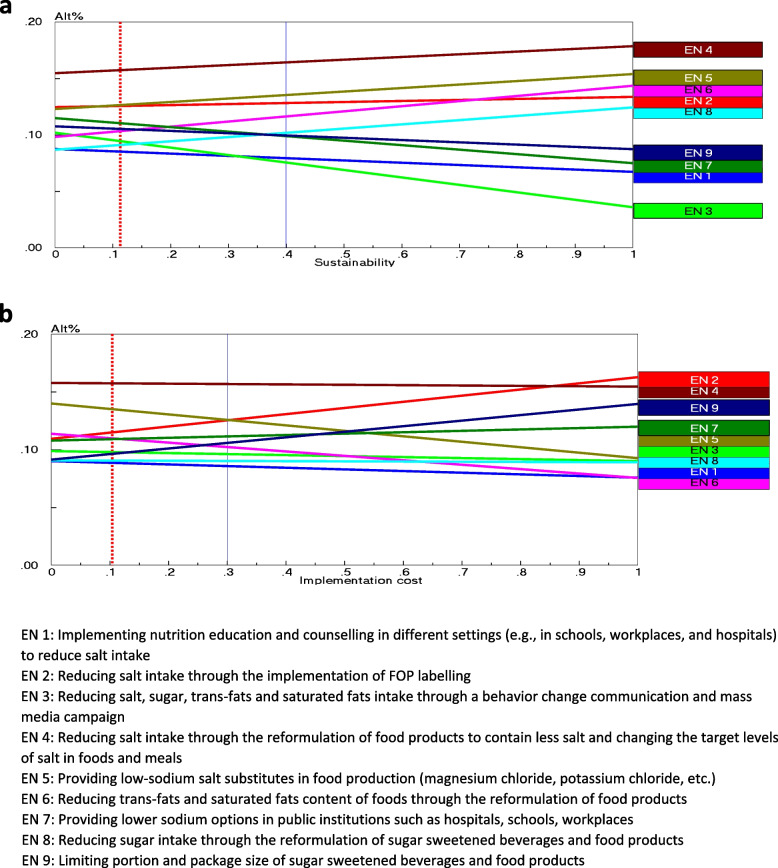
Gradient sensitivity of alternatives when (**a**) sustainability, and (**b**) implication cost criterion is increased

As the gradient sensitivity graph (Fig. 8a) shows, the ranking of alternatives changed when the sustainability criterion increased. Also, an increase in the weight of sustainability criterion (up to 0.4 or 40%) had no impact on the order of the four first alternative preferences.

#### Scenario 3: the sensitivity analysis by slightly altering the weights assigned to ‘implementation cost’

As shown in Fig. 8b, an increase in the weight of implementation costs criterion (up to 0.3 or 30%) had no impact on the order of three alternative preferences.

Accordingly, it may be suggested that the assessment model performed in this paper had an appropriate level of resilience in terms of selecting the best option among the proposed alternatives. Table 5 summarizes the findings of the sensitivity analysis.

**Table 5:** Priorities and rankings of intervention strategies for HTN prevention and control with respect to each scenario

**Alternative **	**Scenario 1**	**Scenario 2**	**Scenario 3**
EN 1	0.088 (9)	0.080 (8)	0.086 (9)
EN 2	0.120 (3)	0.128 (3)	0.125 (3)
EN 3	0.101 (7)	0.076 (9)	0.096 (7)
EN 4	0.152 (1)	0.164 (1)	0.157 (1)
EN 5	0.133 (2)	0.135 (2)	0.126 (2)
EN 6	0.106 (5)	0.116 (4)	0.102 (6)
EN 7	0.108 (4)	0.099 (7)	0.111 (4)
EN 8	0.090 (8)	0.102 (5)	0.090 (8)
EN 9	0.102 (6)	0.100 (6)	0.107 (5)

Data is presented as weight (rank)

EN 1: Implementing nutrition education and counselling in different settings (e.g., in schools, workplaces, and hospitals) to reduce salt intake

EN 2: Reducing salt intake through the implementation of FOP labelling

EN 3: Reducing salt, sugar, trans-fats and saturated fats intake through a behavior change communication and mass media campaign

EN 4: Reformulation of food products to contain less salt and changing the target levels of salt in foods and meals

EN 5: Providing low-sodium salt substitutes in food production (magnesium chloride, potassium chloride, etc.)

EN 6: Reducing trans-fats and saturated fats content of foods through the reformulation of food products

EN 7: Providing lower sodium options in public institutions such as hospitals, schools, workplaces

EN 8: Reducing sugar intake through the reformulation of sugar sweetened beverages and food products

EN 9: Limiting portion and package size of sugar sweetened beverages and food products

### Comparative analysis

In the MCDM approach, the capability and rationality of the proposed methods are proved by comparing them with other stable methods commonly used in the related studies. In this study, based on the relative weights of the evaluation criteria obtained by AHP, the three MCDM tools, including Technique for Order of Preference by Similarity to Ideal Solution (TOPSIS), Simple Additive Weighting (SAW), and Combined Compromise Solution (CoCoSo) were adopted for evaluating the ranking of population-based nutrition interventions. The TOPSIS is one of the well-known classical MCDM methods, in which the alternatives are ranked based on their distance from defined ideal and negative-ideal solutions [81]. The basic concept of the SAW method is to search for the weighted sums obtained from the performance ratings of each alternative on all criteria [82]. The CoCoSo method is based on the integration of SAW and the exponentially weighted product model (MEP) to deduce compromise solutions [83].

**Fig. 9:**
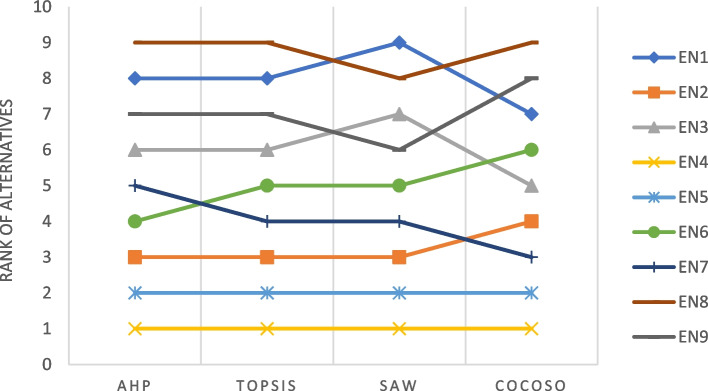
Ranking results by different methods

As Fig. 9 shows, the results obtained by the proposed approach have high degrees of similarity with the results derived by other MCDM methods. In particular, the top three alternatives selected by the proposed method are completely consistent in three methods. In addition, the Spearman’s rank correlation coefficients between the ranking obtained from the proposed model and TOPSIS, SAW, and CoCoSo methods were 0.983, 0.950, and 0.900, respectively. The high Spearman’s rank correlation coefficient demonstrated a very strong relationship between the results of AHP and the MCDM methods considered for the comparative analysis. Overall, it can be concluded that the proposed model is robust and the obtained results are reliable and can be considered as useful guidelines for managers and decision-makers.

## Discussion

In this study, an AHP-Delphi integrated method was proposed to rank and select the most appropriate population-based nutrition interventions pertaining to prevention and control of hypertension from the perspective of researchers, specialists, and experts involved in various stages of health policymaking in Iran.

Two key sets of findings could be drawn from this study. First, it is possible to use an AHP-Delphi framework to generate evidence-informed decision-making in the public health setting. The example of such framework presented in this study demonstrates how it can be used to create a prioritizing of nutritional interventions for control and prevention of hypertension by combining the performance of interventions against a range of criteria. AHP performs this through the powerful combination of decision-makers priorities and rigorous analytical methods. More importantly, using the AHP method allows the number of trade-offs made at one time by an expert in a complex decision setting to be reduced to a choice between only two decision criteria in each pairwise comparison in the AHP. In other words, a strength of the AHP method is the reduced cognitive burden for the experts by decomposing a complex decision problem into a limited number of pairwise comparisons [84, 85].

As far as the researchers of this study investigated, the AHP has not been used to make decisions about population-based nutrition interventions so far. However, it has been applied in numerous fields, including marketing [45], research and development project selection and resource allocation [46], health care and medical decision-making [53, 54], and other decision-making contexts [47, 49–51, 86]. Previous literature includes some reviews of the application of MCDM in the health care sector [54, 86, 87]. Liberatore and Nydick reviewed the application of AHP in health care based on 50 published articles [86]. According to the results of their systematic review, the AHP appears to be a promising support tool for shared decision-making between patient and doctor, evaluation and selection of medical diagnoses and treatments, human resource planning, selection and evaluation of projects and technology in health care setting, evaluation of health care facilities, and health care policy analysis [86]. More recently, Yetim et al. applied the AHP method to determine how policies and measures used in Turkey to address coronavirus disease 2019 (COVID-19) are prioritized by health care professionals and other segments of the community [88].

Secondly, the results of applying the framework represent the experts’ preferences for nutrition-related interventions to combat the growing prevalence of hypertension. In this study, nine interventions were included in the final ranking based on eight assessment criteria. According to the results, ‘reducing salt intake through the reformulation of food products to contain less salt and changing the target levels of salt in foods and meals’ was ranked as the intervention with the highest priority. Based on the evidence, this intervention is one of the most cost-effective and practical actions for the prevention and control of NCDs, so that in 2017, the WHO recommended reduction of sodium intake through food products reformulation and setting salt target levels in foods and meals as a ‘best buy’ intervention [12]. Several previous systematic reviews on policies to improve diets also found that reformulation of food products resulted in a reduction in population-wide salt consumption [89, 90]. A multi-component salt reduction program developed in the United Kingdom starting in 2003 corresponded to a substantial decrease in the sodium content of some food products, and in sodium intakes for the population [91]. This reduction was driven mainly by food product reformulation to reduce the sodium density of foods and, to a smaller extent, by changes in food choices. The intensity of effects was similar across all subgroups defined by their socioeconomic status [92]. As such, reformulation is expected to partially attenuate the existing disparities in health service utilization. However, several design characteristics of such interventions, such as the voluntary or mandatory nature of the program, may influence the impact of the policy [93]. Based on the evidence, the potential health gains are much greater from a legislated approach and engagement with all sectors of the food industry [94, 95]. Some countries such as Argentina and South Africa have extended mandatory limits to enforce salt targets across a broad range of commonly consumed food [96, 97]. Nevertheless, the United Kingdom presents the most successful and comprehensive example of developing voluntary salt target levels for various food product categories, which many other countries follow [16]. Kuwait’s voluntary bread reformulation agreement also achieved significant results in a short time, so that a 20% reduction in the salt content of almost all types of bread occurred by the end of the first year of implementing the intervention [98]. The success of the voluntary approaches depends on strong government leadership, extensive advocacy activities, close collaboration with the industry, proper monitoring of the salt content of the selected foods, and most importantly, publication of the results to hold the food industry to account [99].

However, working with and engaging the food industry to encourage reformulation of food to contain less salt can be challenging. Several arguments, including technical feasibility and consumers’ taste acceptance of lower salt food, may be employed to justify the lack of progress in salt reduction in food by some sections of the food industry. The broad range of salt levels observed in a similar range of already existing food products, many of which are below the target, evidences the technical feasibility of reducing salt levels further in almost all processed food [16]. In terms of taste, the human salt taste receptors can adapt and become more sensitive to low salt concentrations within only 4–6 weeks; therefore, a small gradual decrease in the sodium content of processed food cannot be detected [100, 101]. This means that foods with lower salt content will taste as salty as highly salted foods before the adjustment. Furthermore, evidence indicates that, once salt intake has been reduced, consumers prefer foods with less salt [101]. In the United Kingdom, for example, reducing the salt content of major brand products in supermarkets by 20–30% over 3 years did not affect sales and consumer preference [16].

Based on our results, ‘providing low-sodium salt substitutes in food production (magnesium chloride, potassium chloride, etc.)’ was ranked as the second priority of interventions. Replacement of dietary salt with lower sodium salt substitutes, i.e., salt enriched with potassium or other similar ingredients such as magnesium, is a potential blood pressure-lowering strategy being considered by several countries (e.g., the United States, China, and United Kingdom) and public health organizations [102–104]. This strategy has been effective in Finland, where common salt has been replaced mainly by low-sodium potassium-enriched ‘pansalt’ [105]. In China, replacing salt used in home cooking with a low sodium alternative reduced mean SBP by 5.4 mmHg in the population [104]. Furthermore, in a recent meta-analysis of randomized controlled trials, low sodium salt substitutes, compared with regular salt, reduced average SBP by 7.81 mmHg and DBP by 3.96 mmHg; the effects were similar across hypertensive, normotensive, and mixed populations [106].

The approach to incorporating potassium-enriched salt substitutes as a main non-sodium alternative, into the public health interventions may vary by country depending on the population’s main dietary sodium source. In countries such as the United States, where 70% of the total sodium intake comes from processed foods and restaurant meals, food product reformulation may be a particularly effective sodium reduction strategy [107]. In contrast, substituting regular salt added to food during domestic cooking may be more effective in countries, such as China, where up to three-fourths of the sodium intake comes from salt added during food preparation [108, 109].

A major concern in the implementation of the salt substitution strategy is its acceptability caused by an alteration in flavor and palatability of salt because of the replacement of sodium with potassium as a main non-sodium alternative [110]. To overcome these problems, a limited percentage of KCl has been used in combination with other nutritionally accepted agents (MgCl2, MgSo2, etc.) [111]. In studies exploring taste acceptability of six different potassium-enriched salt substitutes, more than 80% of individuals did not differentiate between regular salt and potassium-enriched salt substitutes containing less than 30% KCl [112]. The other concern related to potassium-enriched salt substitutes is a possible increased risk of hyperkalemia, consequent arrhythmias, and sudden cardiac death, especially among individuals suffering from impaired potassium excretion such as chronic kidney disease [113]. However, there is considerably weak evidence regarding the relationship of potassium-enriched salt with serum potassium levels and the occurrence of hyperkalemia in patients with chronic kidney disease and others at risk for hyperkalemia [114].

In our study, according to the results of AHP, ‘reducing salt intake through the implementation of FOPL’ was identified as the third priority intervention. FOP labeling is considered a cost-effective strategy to empower people to make informed and healthier food choices [115, 116]. As a minimum, many countries have nutrition information tables on the back of food packaging, but the WHO, as well as other international health agencies, have sought to promote FOP labeling strategies as part of the comprehensive policy action in response to the growing prevalence of NCDs [12, 117, 118]. There is variability in labeling systems in use worldwide regarding specific functional and visual characteristics such as the type of expression (including whether voluntary or mandatory), and whether the label gives any interpretive and non-interpretive guidance to the consumer [119]. Whit in some countries FOP labeling schemes are mandatory (e.g., Chile, Thailand, and Finland), although most countries have voluntary systems (e.g., France, South Korea, Australia, and New Zealand) [120]. Interpretive labels present graphics, symbols, or cautionary text to represent the overall healthfulness or nutrient content of a product, such as ‘Chilean style warning labels’ that mark products as high in salt, saturated fats, sugar, or calories; the ‘Nutri-Score label’, as used in France, which presents a color spectrum along with letter grades to provide a summary indicator of product healthiness; ‘Multiple Traffic Light’, used in the United Kingdom and others, indicating red (high), amber (moderate) or green (low) levels of nutrients of concern; and the ‘Health Star Rating’, as used in Australia and New Zealand, which uses a star rating scale of the half to five stars [121]. Noninterpretive FOP labeling scheme, such as the Guideline Daily Amount, displays information only as numbers rather than figures, colors, or symbols, allowing consumers to make their own judgments about the healthfulness of products [122].

Finland was the first country to introduce a mandatory warning label in the early 1990s, requiring that foods with salt content higher than a maximum level must represent a high-salt content warning label, and foods containing a low level of salt are allowed to carry a low-salt label [123]. During the past three decades, the one-third reduction in the Finnish average salt intake has been accompanied by a more than 10-mmHg drop in average population SBP and DBP as a result of systematic action on salt, including the labeling regulations which enable consumers to identify low-salt products [15].

On the other hand, the use of FOP labeling on products may also potentially stimulate food processors to reformulate products to meet nutrition criteria, so that they avoid carrying negative FOP labels [124]. Thus, through reformulation, labeling may benefit all consumers, not just those who read the label. In 2016, Chile launched a comprehensive black warning logo program for products that exceed limits of sodium, saturated fats, total sugars, and total energy (kilocalories) [125]. A comparison of the nutritional profiles of food products before and after the first year of Chilean FOP labeling showed a significant decrease in the proportion of products that should carry sodium warning labels, indicating that companies reformulated products to avoid the FOP warning label requirements [126]. A recent meta-analysis also indicated that food labelling significantly reduced the product contents of sodium by 8.9% (95% CI = − 17.3, − 0.6%) and artificial trans-fat by 64.3% (95% CI = − 91.1, − 37.5%) [124].

In Iran, in line with the WHO’s global targets to reduce the burden of NCDs and recommended policies for the prevention and control of NCDs in 2015, MoHME established the national NCD committee intending to integrate all decisions and activities and make evidence-based policies for tackling NCDs [18]. In 2016, Iran’s National Action plan for the prevention and control of NCDs was developed, considering several WHO’s recommended interventions to reduce the mortality rate of NCDs by 30% until 2030 [18]. The primary approved strategy to decrease unhealthy diet was setting a maximum level of salt, sugar, and saturated and trans fatty acids in food in the national regulations for staple foods such as bread, dairy and oil products. Furthermore, nutritional traffic light labeling on food packages was established to signify the salt, sugar, and saturated and trans fatty acids content of the foodstuff [127]. Despite the recent efforts of MoHME and the Food and Drug Administration, as a member of the national NCD committee, formulation and implementation of those policies have encountered some challenges [128, 129]. One of the most prevailing issues is a research gap on systematic surveillance and process, or impact of evaluation of the current policy to assess progress and guide further efforts [127, 129]. Although the maximum permitted levels of salt and saturated and trans fatty acids in food in the national regulations were modified, those standards are still far from WHO recommendations [18]. For example, the maximum permitted percentages for trans and saturated fats were set as 2–5% and 30–65% of edible oils and fats, respectively, that exceed the WHO recommendations [12, 18, 130]. A recent policy analysis study in Iran highlighted the most essential challenges of nutrition food labeling scheme, including poor media involvement in public awareness of nutrition traffic light labeling, inconsistency of traffic light colors with nature of some food products, inconsistency of traffic light color ranking with other food-related standard guidelines, and lack of a comprehensive evaluation plan [129]. In the study by Haghighian et al., insufficient knowledge in interpreting the labels, the small sizes of the traffic light labels on the back of packages, and the lack of substitutes for food products with red traffic light signs were the main challenges of consumers encounter with such labels [131]. Another challenge of implementing the aforementioned regulatory programs is the discrepancy between the content of target nutrients in pre-packaged foods based on test results and the value stated on the nutrition labels [132, 133]. In the research by Ghazavi et al., the discrepancy of the actual trans fatty acids content based on chemical analysis with value declared on the traffic light labels was observed in more than 80% of Iranian sweets [128]. This evidence along with the growing prevalence of hypertension in Iran [56] suggests that revision of existing policies and development of new prioritized strategies using MCDM methods can lead to meaningful outcomes for the prevention and control pf NCDs.

An overview of the Iranian government and political system reveals that officials and authorities of most governmental institutions change every 4 years following the change of the head of the executive branch of a state, which may affect the sustainability of the implementation of approved programs (e.g., health programs, etc.). Furthermore, the cost of implementing the programs is a key factor in the successful implementation and effectiveness of health programs, especially in Iran, due to the recently imposed economic sanctions. As a result, we conducted a sensitivity analysis to determine the impact of changes in the weights assigned to sustainability and implementation cost criterion on the final ranking of the alternatives. Also, we performed comparisons with selected other MCDM methods and tested the robustness of the suggested model. According to these, the results obtained from proposed model have sufficient validity and reliability to be a useful guideline for health care professionals and policy-makers.

### Limitations and strengths

As far as the researchers of the current study investigated, this was the first research that implemented a comparative analysis for prioritizing population-based nutrition interventions pertaining to prevention and control of hypertension using an AHP-Delphi integrated framework. Also, the multi-stakeholder approach used in this study, which helped to extract the diverse perspectives of researchers and specialists, as well as experts involved in different stages of health policy-making, were the main strengths of the present study.

However, several limitations should also be addressed. The first limitation is related to methodological approach. In the AHP method, weights for decision criteria and alternatives are assumed to be independent, but this may not be the case. Furthermore, rank reversal might also be possible if an alternative is deleted or added to the set of alternatives used for evaluation. Regarding the pairwise comparisons in AHP, when the number of criteria or alternatives increases, the AHP questionnaire content becomes large in quantity, and the pairwise comparisons become confusing. As a result, a high level of inconsistency is expected. Therefore, the comparisons might be returned to the participant for several times for improvement aims. Thus, our analysis was restricted to a limited number of criteria and alternatives. Another limitation is that study analysis uses the preferences of experts involved in policy-making, and researchers to define and weight criteria and alternatives. It is often argued that public preferences should also be employed in policies and programs selection and resources allocation [134].

### Future directions

As future directions, other new multicriteria evaluation methods (BWM, FUCOM, LBWA, etc.) and various hybrid of them could be applied in the selection of nutritional interventions to combat hypertension, and the findings could be compared. Also, this will help in selecting the most appropriate method for a given health decision-making context (e.g., low- and middle-income countries with limited resources), taking practical aspects into account. While we used AHP to facilitate group decision-making regarding the prioritization of population-based nutritional intervention, in future research the decision objective could certainly be modified to address other group decisions such as the evaluation and selection of medical treatments. Furthermore, this study can be expanded to other public health problems such as obesity, diabetes, etc., to promote public health status and mitigate the rising burden of disease. While the results of AHP only apply to the particular decision context we specified, they are not meant to be generalizable. However, the presented approach can be applied to anywhere by modifying the indicators and repeating the assessment of experts in different countries. As a result, the model can provide measurement tools to support decision makers in different communities.

### Managerial implication

The current study documented the utility of MCDM methods for prioritizing nutrition-related interventions to combat the growing prevalence of hypertension in Iran. Although several efforts to tackle hypertension have been implemented, evidence have shown that the rates of prevention, and control of high blood pressure remains unsatisfactory. it is necessary for Iranian policy-makers to assess the root cause of the policy formulation challenges and develop targeted solutions to address each them in collaboration with other stakeholders and empowered parties. More important, prioritizing strategies through a collaborative decision-making process, where key stakeholders consider all the available intelligence on the known strategy options to address health problem and all criteria for assessing them in accordance with the current socioeconomic status of population can help to develop more appropriate actions and make more rational resource allocation.

In this study an effective approach for selecting the appropriate nutritional interventions pertaining prevention and control of hypertension has been stablished. By conducting the Delphi-AHP integrated model, participants in our survey seemed to view reformulation of food products to contain less salt as being a more prioritized strategy for tackling hypertension. The strategy of providing low-sodium salt substitutes in food production was also highly ranked as a nutrition-related strategy. Using MCDM models provides a basis for informed decision-making to save costs and resources in the planning phase of population-based health interventions. Since the decision support tool used in the study can be applied anywhere, this study can be a helpful guide for researchers, and decision-makers in other communities in developing suitable and effective strategies for prevention and control of hypertension. Furthermore, the proposed approach can be replicated for similar health problems (such as obesity, smoking, low physical activity, etc.) with appropriate modification of criteria and substitutions needed to meet real conditions.

For the successful implementation of the proposed intervention, decision-makers must improve inter-sectoral collaboration between the health and education sectors, the media, food producers, and food industries. All relevant stakeholders must be involved in policy process, and all policy actors should be given opportunities to state their opinions on the policy. Finally, most reviewed programs in Iran lack proper monitoring and evaluation scheme. It is necessary to consider strategies for systematic monitoring, continuous policy evaluation, and documenting evidence in the whole procedure, such as policy design and implementation, to identify potential barriers and problems, and assess program effectiveness.

## Conclusion

We demonstrated the usefulness of the integrated Delphi-AHP method in prioritizing and selecting public health interventions and provided a guide on how to use it. The findings of this study provide a preliminary evidence base to guide future decisions and directions in tackling hypertension and other risk factors of NCDs through selection of appropriate population-based nutritional interventions. It is hoped that decision-makers and health care managers would consider the recommendations made in this study regarding the current and future health policy in an effort to accelerate advance towards sustainable management of NCDs.

## Data Availability

The datasets used and/or analyzed during the current study are available from the corresponding author on reasonable request.

## Abbreviations

NCDs: Non-communicable diseases
MCDM: Multi-criteria decision-making
AHP: Analytic Hierarchy Process
FOPL: Front-of-package labeling
SBP: Systolic blood pressure
DBP: Diastolic blood pressure
CR: Consistency ratio
